# *RAB32* ‐Linked Parkinson's Disease: Deep Phenotyping, MDSGene Literature Review, and Application of SynNeurGe Criteria

**DOI:** 10.1002/mds.70037

**Published:** 2025-10-17

**Authors:** Teresa Kleinz, Francesco Cavallieri, Max Borsche, Giulia Toschi, Franco Valzania, Valentina Fioravanti, Enza Maria Valente, Pierfrancesco Mitrotti, Micol Avenali, Simone Zittel, Rommi Born, Michele Matarazzo, Alessio Di Fonzo, Edoardo Monfrini, Mandy Radefeldt, Letizia Santinelli, Norman Griebner, Cholpon Shambetova, Max Brand, Carolin Gabbert, Cornelis Blauwendraat, Joanne Trinh, Katja Lohmann, Christian Beetz, Peter Bauer, Norbert Brüggemann, Global Parkinson's Genetics Program (GP2), Christine Klein

**Affiliations:** ^1^ Institute of Neurogenetics, University of Lübeck Lübeck Germany; ^2^ Section for Movement Disorders, Department of Neurology University Hospital of Schleswig‐Holstein Lübeck Germany; ^3^ Neuromotor and Rehabilitation Department, Neurology Unit Azienda USL‐IRCCS Di Reggio Emilia Reggio Emilia Italy; ^4^ IRCCS C. Mondino Foundation Pavia Italy; ^5^ Department of Molecular Medicine University of Pavia Pavia Italy; ^6^ Department of Brain and Behavioral Sciences University of Pavia Pavia Italy; ^7^ Department of Neurology University Medical Center Hamburg‐Eppendorf Hamburg Germany; ^8^ Klinikum Hanau Department of Neurology Hanau Germany; ^9^ Centro Integral de Neurociencias Abarca Campal, Hospital Universitario HM Puerta Del Sur, HM Hospitales Madrid Spain; ^10^ IRCCS Foundation Ca' Granda Ospedale Maggiore Policlinico, Dino Ferrari Center, Neuroscience Section, Department of Pathophysiology and Transplantation University of Milan Milan Italy; ^11^ Centogene Rostock Germany; ^12^ Center for Alzheimer's and Related Dementias (CARD), National Institute on Aging and National Institute of Neurological Disorders and Stroke, National Institutes of Health Bethesda Maryland USA; ^13^ Laboratory of Neurogenetics, National Institute on Aging, National Institutes of Health Bethesda Maryland USA

**Keywords:** genotype–phenotype relationship, monogenic PD, Parkinson's disease, RAB32, seed amplification assay (SAA), SynNeurGe criteria

## Abstract

**Background:**

The *RAB32* p.Ser71Arg variant is a novel cause of monogenic Parkinson's disease (PD), for which detailed phenotypic information is currently scarce.

**Objectives:**

Our aim was to clinically and biologically characterize individuals with PARK‐*RAB32* to gain insights into genotype–phenotype relationships, disease severity, and underlying pathology.

**Methods:**

We conducted a literature review following the MDSGene protocol, alongside detailed phenotyping of 11 PARK‐*RAB32* patients and one prodromal individual from the Rostock International PD (ROPAD) study. In addition to comprehensive scale‐based assessments, including olfactory testing, we obtained neuroimaging data and various biomaterials, and performed α‐synuclein seeding assays (SAA) in cerebrospinal fluid in a subset.

**Results:**

83 patients (72 from the literature) were included in the analysis. The median age at onset was 54 (IQR: 46–61) years. Typical parkinsonism with a favorable dopaminergic response was observed in all patients.

In our cohort, after a median disease duration of 11 years (IQR: 7–19.5), the mean Movement Disorders Society Modified Unified Parkinson's Disease Rating Scale (MDS‐UPDRS) III score was 38.5 ± 21.8 points. Targeted testing revealed autonomic symptoms were present in all individuals, and 10 of 11 patients had hyposmia. Misfolded α‐synuclein was identified in 2 of 2 patients, but not in the prodromal individual. 123I‐FP‐CIT imaging was available for eight patients, revealing neurodegeneration in all of them.

**Conclusion:**

While PARK‐*RAB32* is clinically and likely pathologically similar to idiopathic PD, our study underscores the importance of carefully assessing non‐motor symptoms in this newly described form of PD. According to SynNeurGe criteria, PARK‐*RAB32* is classified as **S** ^+^ (evidence of synucleinopathy), **N** ^+^ (neurodegeneration supported by imaging data), and **G**_P_^+^ (presence of a genetic variant). © 2025 The Author(s). *Movement Disorders* published by Wiley Periodicals LLC on behalf of International Parkinson and Movement Disorder Society.

The recent discovery of the pathogenic variant p.Ser71Arg in the *RAB32* gene as a cause of Parkinson's disease (PD)^1^, ^2^ is the first newly established monogenic form of PD in the last decade. Given their rarity and the present lack of deep phenotyping data, little is known about the distinct clinical and genetic characteristics of patients with PARK‐*RAB32*, disease‐related biomarkers, and the course of the disease.^1^, ^2^, ^3^, ^4^, ^5^, ^6^, ^7^ The p.Ser71Arg founder variant in *RAB32* leads to overactivity of the leucine‐rich repeat kinase 2 (LRRK2), encoded by a gene in which pathogenic variants were identified as the most frequent cause of monogenic PD.^8^, ^9^, ^10^ As in PARK‐*SNCA*, PARK‐*LRRK2*, and PARK‐*VPS35*, the mode of inheritance in PARK‐*RAB32* is autosomal dominant, with evidence of reduced penetrance.^1^

Here, we provide deep phenotyping data, including a comprehensive assessment of non‐motor symptoms, of 11 PARK‐*RAB32* PD patients and one non‐manifesting carrier of the *RAB32* p.Ser71Arg variant, including magnetic resonance (MR) imaging and dopamine transporter scan (DaTSCAN) data for a subset, and compare these findings with 72 *RAB32*‐linked PD patients published to date by applying the standardized MDSGene protocol.^11^, ^12^

2024 marks the first attempts to classify PD biologically,^13^, ^14^ with the main aim to target the molecular basis of PD even before its clinical manifestation. The SynNeurGe criteria that are based on the presence of α‐synuclein, genetic status, and neurodegeneration, also embrace monogenic forms of PD.^13^ Given that synuclein is often not detected in PARK‐*LRRK2*, which has a comparable mechanism, and that α‐synuclein was not found in the single available autopsy report of a patient with PARK‐*RAB32*,^1^ we partially fill this gap here by including the first cerebrospinal fluid (CSF)‐based α‐synuclein amplification assay (SAA) data and extending the SynNeurGe criteria to PARK‐*RAB32*.

## Patients and Methods

### Setting Up and Deep Phenotyping of the PARK‐ *RAB32* Cohort

Eight patients were identified within the Rostock International PD (ROPAD) Study,^15^ with the cohort expanded by recruiting additional family members. Phenotyping was conducted at the Institute of Neurogenetics and Department of Neurology, University of Lübeck, Germany, or off‐site when patients were unable to travel. The Ethics Committee of the University of Lübeck (Ratzeburger Allee 160, Building 70, 23562 Lübeck) approved the study (2023‐105), and we obtained written informed consent from all examined participants. The diagnosis of PD was established using the Movement Disorders Society criteria.^16^ All participants underwent a detailed history taking, including family history, and a videotaped movement disorder examination, which was all conducted by the same movement disorder specialist (both on‐site and off‐site). Two experienced movement disorder specialists rated the clinical‐neurological assessment, blinded to the genetic status. For this purpose, videos of healthy individuals, individuals with idiopathic PD, patients with PARK‐*RAB32*, and first‐degree relatives who were or were not carrying the p.Ser71Arg variant were used. The Hoehn and Yahr scale (H&Y)^17^ and the Movement Disorder Society‐Unified Parkinson's Disease Rating Scale (MDS‐UPDRS I–IV)^18^ were used to assess disease severity, while the Montreal Cognitive Assessment (MoCA)^19^ was used to identify (mild) cognitive impairment with a cutoff score of 26 points. The State–Trait Anxiety Inventory (STAI)‐Y1 and ‐Y2^20^ score (cutoff‐value for anxiety: ≥40 points), the Geriatric Depression Scale (GDS) (cutoff‐value for depression: ≥5 points),^21^ the rapid eye movement (REM) Sleep Behavior Disorder Screening Questionnaire (RBDSQ) cutoff‐value for RBD: ≥5 points),^22^ the Epworth Sleepiness Scale (ESS; cutoff‐value for excessive daytime sleepiness: ≥ 5 points),^23^ and the Questionnaire for impulsive‐compulsive disorders in Parkinson's Disease–Rating Scale (QUIP‐RS)^24^ were administered to detect other non‐motor symptoms such as anxiety, depression, RBD, excessive daytime sleepiness, or impulse control disorders. Autonomic symptoms were evaluated using the Scales for Outcomes in Parkinson's Disease‐Autonomic Dysfunction (SCOPA‐AUT).^25^ Hyposmia was assessed using the University of Pennsylvania Smell Identification Test (UPSIT),^26^ with percentiles determined using the Brumm *et al*.^27^ 2023 update for individuals >50 years of age. Individuals with a score below the 10th percentile were considered hyposmic.

Seven participants underwent structural brain MRI to exclude concurrent brain lesions. As part of routine clinical care, FP‐CIT single photon emission computed tomographies (SPECTs) were performed in eight patients. The FP‐CIT SPECT images were contributed by two different centers and acquired 4 to 5 hours after intravenous administration of 123I‐FP‐CIT (185 MBq DaTSCAN), which was injected 60 minutes after thyroid blockade with sodium perchlorate. All scans were evaluated through visual inspection as well as a semi‐quantitative analysis using volumes of interest (VOIs), with the occipital cortex (OC) serving as the reference region for FP‐CIT binding. Specific binding ratios were calculated for the entire striatum (Str/OC), the head of the caudate nucleus (Caud/OC), the whole putamen (Put/OC), and both the anterior (aPut/OC) and posterior (pPut/OC) segments of the putamen. Analyses were conducted separately for the left and right hemispheres.

### Genetic Testing, Haplotyping, and SAA

The patients were previously enrolled in the ROPAD study^8^ and identified among a subset of 3,354 patients with available genome sequencing data. This subset was selected for genome sequencing based on their early age at onset (AAO) and/or a positive family history, and tested negative for disease‐causing variants of 68 other genes related to PD or parkinsonism and was screened for the *RAB32* p.Ser71Arg variant.^12^ Positive findings were confirmed by Sanger sequencing.

Haplotype analyses were performed using genome data and confirmed with long‐range polymerase chain reaction, sequencing, and phasing by “genome walking”.^28^ Genetic ancestry was determined from whole‐genome sequencing data by characteristic genotype‐based clustering as described in Westenberger *et al*.^8^ Furthermore, a range of biomaterials was obtained, including serum, plasma, and CSF in a subset. The SAA to detect α‐synuclein seed amplification in CSF was performed as described^29^ at Amprion.

### Systematic MDSGene Literature Review

We performed a literature search on the English‐language literature using the PubMed database (https://pubmed.ncbi.nlm.nih.gov/), according to the standardized extraction protocol of MDSGene,^11^ and compared phenotypic descriptions of PD patients with individual‐level data from previous publications, including published Supplementary data, with the data from our PARK‐*RAB32* cohort. The exact search term can be found in Table S1. Until March 24, 2025, seven papers in the literature reported patients with PARK‐*RAB32*.^1^, ^2^, ^3^, ^4^, ^5^, ^6^, ^7^ Here, we refer to the most recent or comprehensive descriptions of duplicate reports (excluding a total of 18 redundant descriptions). Seven patients from our cohort were also previously reported,^3^, ^6^ but we add deep‐phenotyping, imaging, and SAA data.

## Results

### Literature Review and Clinical Comparison to Our PARK‐ *RAB32* Cohort

In the following, data from a combined study group of 83 PARK‐*RAB32* patients (72 from the literature and 11 from our cohort) are presented. The median AAO was 54 years (IQR: 46–61; range: 31–82 years; missing data: 2.4%), the median age at examination (AAE) 70 years (IQR: 60.5–78; range: 44–98 years; missing data: 54.2%), and the median disease duration 14 years (IQR; 10–21; range: 0–39 years; missing data: 21.7%). Forty‐six (55.4%) were women. Of the patients with (self‐reported) ancestry information available (n = 65; 78.3%), 87.7% were of European ancestry, 9.2% were of Tunisian ancestry, and 3% of mixed ethnicity (Fig. S1). A positive family history was reported in 74% of the patients with information available (n = 77; 92.8%).

**FIG. 1:**
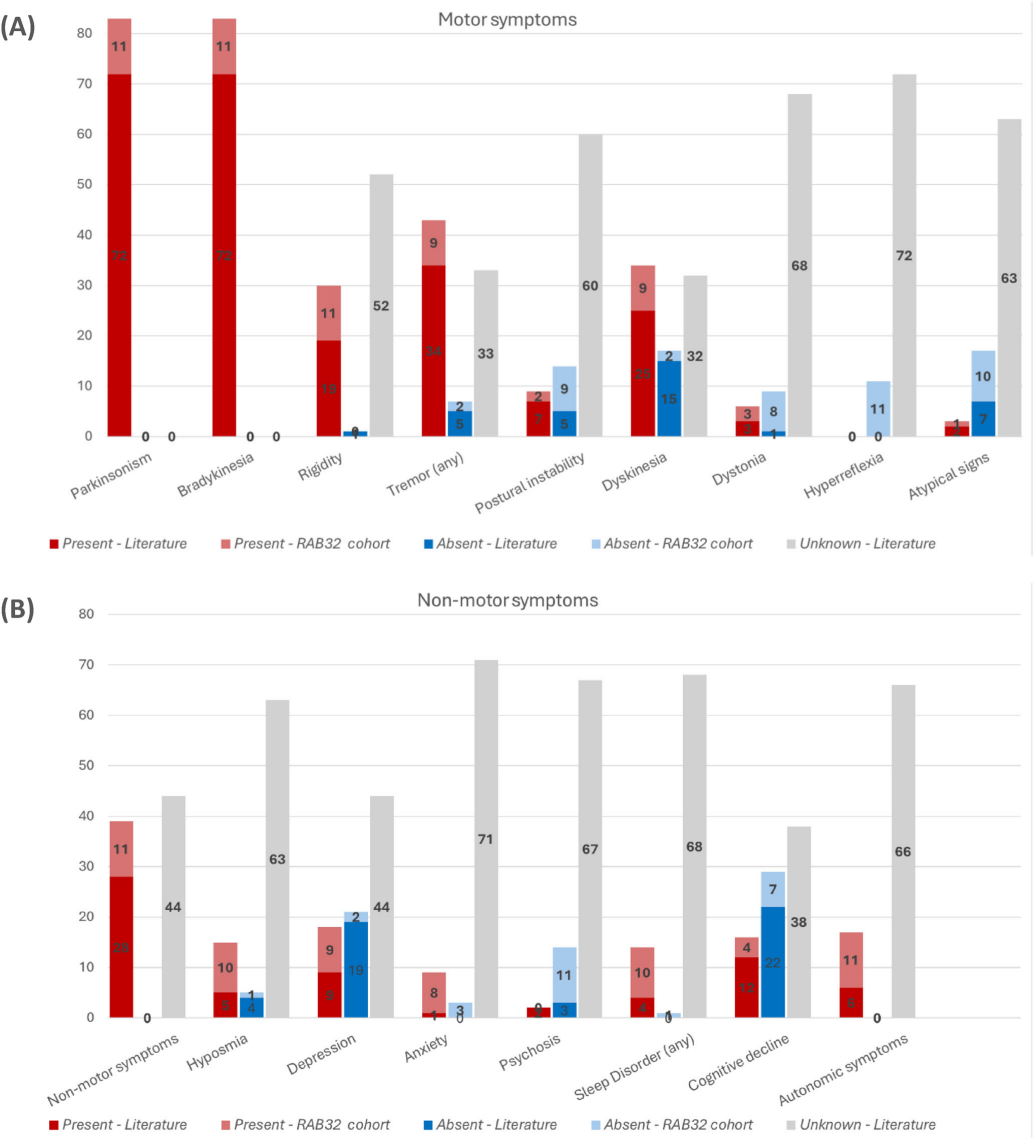
Presence of motor (A) and non‐motor symptoms (B) in *RAB32*‐PD, reported in the literature (dark red or blue parts of the stacked bar diagram) and observed in our PARK‐*RAB32* cohort (light red or blue parts of the stacked bar diagram). [Color figure can be viewed at wileyonlinelibrary.com]

All included patients had classic parkinsonism with a good response to dopaminergic medication, while only one patient developed camptocormia and antecollis within the first 10 years of disease onset (Fig. 1A). Detailed motor evaluation (H&Y, MDS‐UPDRS III), as well as non‐motor symptoms, were not reported in the literature in up to 98.6% of the patients (Fig. 1B). Most frequently reported was cognitive impairment, which was present in 35.3% of patients according to the literature (Table S4).

### Demographic, Clinical, and Genetic Data in Our PARK‐ *RAB32* Cohort

**FIG. 2:**
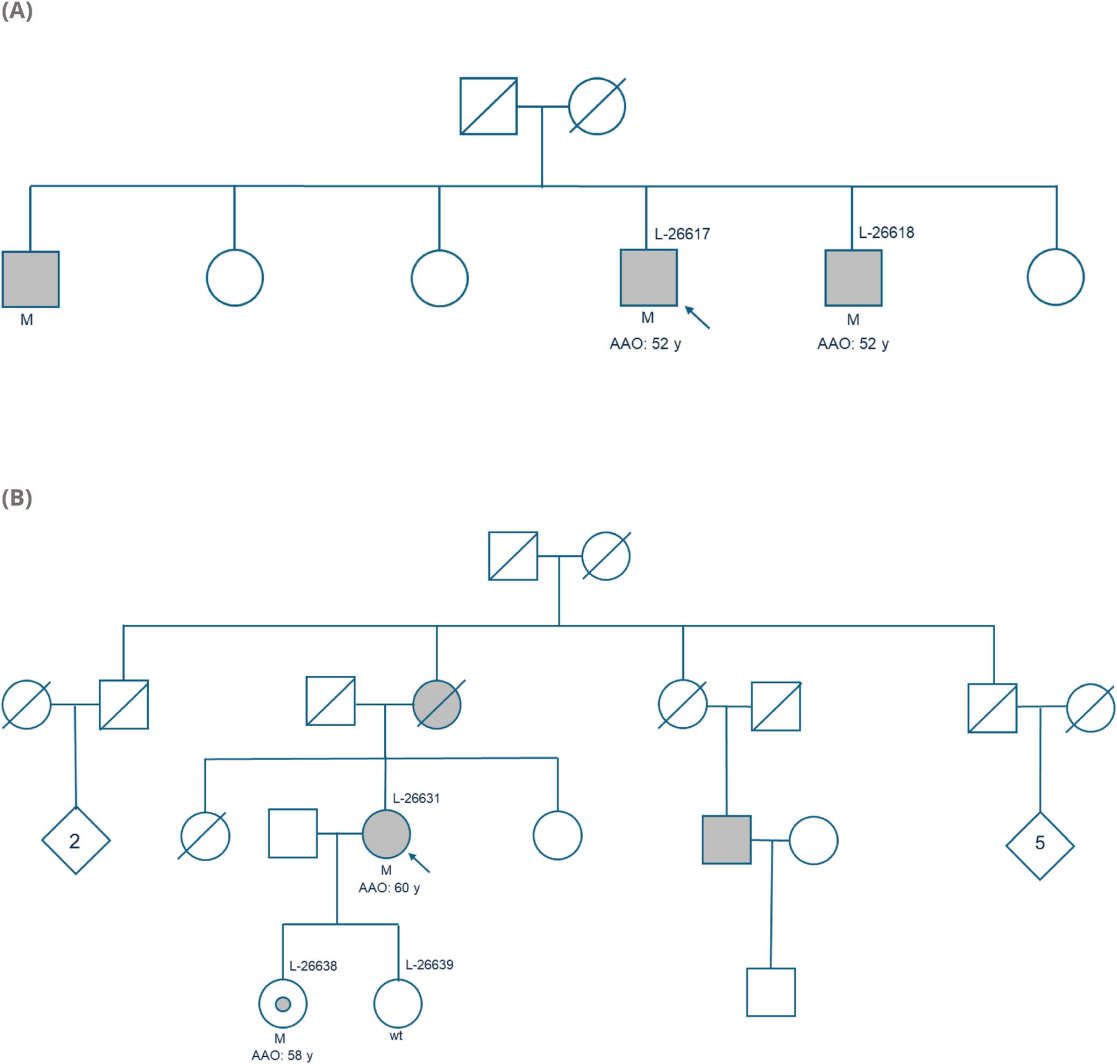
Pedigrees of *RAB32* families. The first family, ITA‐I, from Italy (A), comprises three brothers affected by *RAB32*‐PD. Otherwise, the family history is blank. Both parents died in their late 80s. Two of the brothers were examined in this study (L‐26617 and L‐26618). The second family, GER‐I, originates from Germany (B). The index patient (L‐26631) and both daughters, L‐26638 (*RAB32* p.Ser71Arg carrier with subtle signs) and L‐26639 (wt), were examined. The patient's mother and cousin from the maternal side are also affected by PD. PD, Parkinson's disease; M, confirmed mutant; wt, wildtype; AAO, age at onset; y, years. [Color figure can be viewed at wileyonlinelibrary.com]

From August 2024 to January 2025, we included 12 (10 women, 83%) individuals from 10 distinct families with a heterozygous *RAB32* p.Ser71Arg pathogenic variant in our deeply phenotyped cohort. Eleven of them had a PD diagnosis, and one individual (who was the daughter of one of the patients) showed subtle motor signs compatible with PD, but insufficient to establish a clinical PD diagnosis (Videos [Link], [Link]). Additionally, we examined two sons of affected (female) PD patients who did not carry the pathogenic variant (Table S2). Seven of 11 (63.6%) patients had a positive family history. Figure 2 shows two exemplary pedigrees displaying reduced penetrance (Fig. 2A; Fig. S2). Nine patients were of Italian descent, one was Armenian, and one patient and her daughter originated from Germany. All were of (self‐reported), and, in all of five individuals, genetically confirmed European ancestry (based on the genome data) (Table S3), and all shared the same ancestral founder haplotype.

After a median disease duration of 11 years (IQR: 7–19.5), the median H&Y score in our cohort was 3 (IQR: 2–3) points, the mean MDS‐UPDRS III score was 38.5 ± 21.8 points (*on*‐med), and the mean MoCA score was 23.2 ± 6.1 points, with seven patients falling below the cutoff score of 26 points (Tables 1 and 2, Tables S4). All patients self‐reported motor fluctuations, whereas the mean MDS‐UPDRS IV score showed 7.6 ± 6 points. Nine of 11 (91.8%) patients had excessive daytime sleepiness on ESS screening; 5/11 (45.5%) showed RBD, based on the applied questionnaire, and eight of 11 (72.7%) rated above the cutoff for depression, according to the GDS. Hyposmia, scoring below the 10th UPSIT percentile, was detected in 10 of 11 (90.9%) patients (more detailed individual‐level data are available in Table 2).

**TABLE 1:** Demographic and clinical aspects of the included RAB32 individuals.

Family ID	Local ID	Sex	AAE (y)	AAO (y)	DD (y)	H/Y	MDS‐UPDRS III	MoCA	Seeding [S]	N within imaging^a^ [N]
Patients with PD										
ITA‐I	L‐26617	M	64	52	12	2	39	23	Yes	Yes
ITA‐I	L‐26618	M	59	52	7	2	25	28	N/A	Yes
GER‐I	L‐26631	F	83	60	23	5	61	17	N/A	N/A
ITA‐II	L‐26755	F	74	57	17	3	56	10	N/A	N/A
ITA‐III	L‐26753	F	55	48	7	2	17	25	Yes	N/A
ARM‐I	L‐26809	F	66	58	8	2.5	12	25	N/A	Yes
ITA‐IV	L‐26914	F	77	66	11	3	36	27	N/A	Yes
ITA‐V	L‐26915	F	78	56	22	5	87	17	N/A	N/A
ITA‐VI	L‐26934	F	63	60	3	2	31	30	N/A	Yes
ITA‐VII	L‐26913	F	53	46	7	3	34	28	N/A	Yes
ESP‐I	L‐27021	F	71	49	22	3	26	25	N/A	N/A
*RAB32* individual with subtle motor signs										
GER‐I	L‐26638	F	58	N/A	N/A	1	6	30	No	N/A

*Note*: All enrolled participants were heterozygous for the pathogenic variant p.Ser71Arg [G].

Abbreviations: PD, Parkinson's disease; M, male; F, female; AAE, age at examination; AAO, age at onset; DD, disease duration; H/Y, modified Hoehn and Yahr scale; MDS‐UPDRS III, Movement Disorder Society‐Unified Parkinson's Disease Rating Scale III; MoCA, Montreal Cognitive Assessment; [S], α‐synuclein pathology; N, neurodegeneration; [N], neurodegeneration evidence; N/A, not applicable; [G], pathogenic gene variant.

^a^ DaTScan images used.

**TABLE 2:** Clinical signs and symptoms of the examined individuals

Individual local ID	L‐26617	L‐26618	L‐26631	L‐26755	L‐26753	L‐26809	L‐26914	L‐26915	L‐26934	L‐26913	L‐27021	L‐26638
Sex	M	M	F	F	F	F	F	F	F	F	F	F
AAO; DD (y)	52; 12	52; 7	60; 23	57; 17	48; 7	58; 8	66; 11	56; 22	60; 3	46; 7	49; 22	N/A
Motor												
Parkinsonism	Yes	Yes	Yes	Yes	Yes	Yes	Yes	Yes	Yes	Yes	Yes	No
Bradykinesia	Yes	Yes	Yes	Yes	Yes	Yes	Yes	Yes	Yes	Yes	Yes	Yes
Tremor	Yes	Yes	Yes	Yes	Yes	Yes	Yes	Yes	Yes	Yes	Yes	No
Rigidity	Yes	Yes	Yes	Yes	Yes	Yes	Yes	Yes	Yes	Yes	Yes	No
Postural instability	No	No	Yes	No	No	No	No	Yes	No	No	No	No
H/Y	2	2	5	3	2	2.5	3	5	2	3	3	N/A
MDS‐UPDRS III	39	25	61	56	17	12	36	87	31	34	26	4
Dyskinesia	M.I.	M.I.	M.I.	M.I.	M.I.	M.I.	No	M.I.	No	M.I.	M.I.	No
Dystonia	No	No	No	No	No	No	Yes	Yes	No	No	Yes	No
Hyperreflexia	No	No	No	No	No	No	No	No	No	No	No	No
Atypical	No	No	No	No	No	No	Yes*	No	No	No	No	No
Diurnal fluctuations	Yes	Yes	Yes	Yes	Yes	Yes	Yes	Yes	Yes	Yes	Yes	No
Levodopa response	Good	Good	Good	Good	Good	Good	Good	Good	Good	Good	Good	N/A
Motor fluctuations	Yes	Yes	Yes	Yes	Yes	Yes	Yes	Yes	Yes	Yes	Yes	No
MDS‐UPDRS‐IV	9	7	12	16	5	0	0	17	0	9	9	N/A
Non‐motor												
Olfactory dysfunction	Yes	Yes	Yes	Yes	Yes	Yes	No	Yes	Yes	Yes	Yes	No
UPSIT score	10	24	9	12	29	18	28	15	23	17	15	36
UPSIT percentile [%]	**1**	**7**	**1**	**2**	**5.5**	**5**	25	**4**	**5**	**1**	**4**	62.5
Depression	Yes	No	Yes	Yes	Yes	Yes	Yes	Yes	No	Yes	Yes	No
GDS score	4	1	**9**	**9**	**8**	**8**	**6**	**6**	0	**8**	**7**	3
Anxiety	Yes	No	No	Yes	Yes	Yes	No	Yes	Yes	Yes	No	Yes
STAI‐Y1	**42**	40	**41**	**51**	**46**	36	36	**47**	**50**	**41**	22	**46**
STAI‐Y2	**46**	**44**	**53**	**49**	**46**	**48**	**41**	37	**47**	39	22	**50**
Psychotic	No	No	No	No	No	No	No	No	No	No	No	No
Cognitive decline	Yes	No	Yes	Yes	Yes	Yes	No	Yes	No	No	Yes	No
MoCA	**23**	28	**17**	**10**	**25**	**25**	27	**17**	30	28	**25**	30
Sleep disorder	Yes	Yes	Yes	Yes	Yes	Yes	Yes	Yes	Yes	Yes	No	Yes
RBDSQ	3	**11**	**10**	**12**	4	**10**	0	**7**	1	3	3	**5**
ESS	**14**	**6**	**17**	**7**	**15**	3	**6**	**7**	**10**	**10**	2	**10**
Autonomic	Yes	Yes	Yes	Yes	Yes	Yes	Yes	Yes	Yes	Yes	Yes	Yes
SCOPA‐AUT	23	24	21	33	8	36	6	21	10	7	26	5

*Note*: **Bold values**, above cutoff within scores.

Abbreviations: M, male; F, female; AAO, age at onset; DD, disease duration; H/Y, modified Hoehn and Yahr scale; MDS‐UPDRS, Movement Disorder Society‐Unified Parkinson's Disease Rating Scale; UPSIT, University of Pennsylvania Smell Identification Test; GDS, Geriatric Depression Scale; STAI, State‐Trait Anxiety Inventory; MoCA, Montreal Cognitive Assessment; RBDSQ, REM sleep behavior disorder screening questionnaire; ESS, Epworth Sleepiness Scale; SCOPA‐AUT, Scale for Outcomes in Parkinson's disease for Autonomic symptoms; N/A, not applicable; M.I., medication‐induced.

The individual with the *RAB32* pathogenic variant without an established PD diagnosis showed mild hypomimia and lateralized bradykinesia, including reduced arm swing and delayed shoulder shrug on the right side. In addition, depressive symptoms and constipation were reported to have been present for some years. Furthermore, RBD and excessive daytime sleepiness were identified (Table S4). Examples of videotaped examinations are provided in the Supplementary Material.

### SAA, Neuroimaging, and SynNeurGe Criteria

CSF SAA revealed the presence of α‐synuclein pathology in 2/2 *RAB32*‐linked PD patients who donated CSF, whereas the individual with subtle signs of PD had a negative α‐synuclein SAA result.

DaTSCAN results were available for 7/11 patients and confirmed neurodegeneration in all (Table 1, Fig. S3). The combined qualitative and quantitative analyses confirmed asymmetric tracer binding and a rostro‐caudal gradient in six patients, consistent with the clinical picture of lateralized parkinsonism and the absence of significant asymmetry in one patient. Structural brain MRI was carried out in seven individuals and ruled out concurrent brain lesions (Table S5).

Based on the SynNeurGe criteria, the biological status of the patients with available data was synucleinopathy (**S** ^+^), neurodegeneration (**N** ^+^), and strong genetic predisposition with incomplete penetrance (**G**_P_^+^).

## Discussion

For the first time, PARK‐*RAB32* patients were classified according to the SynNeurGe classification criteria, including the evidence of synucleinopathy (**S** ^+^), neurodegeneration based on imaging data (**N** ^+^), presence of a genetic PD variant (**G**_P_^+^), in conjunction with the clinical picture of PD (**C** ^+^).^13^ Our findings also serve as first evidence of the presence of misfolded α‐synuclein in patients with PARK‐*RAB32*, whereas the available single autopsy report from the literature described sparse neurofibrillary tangle pathology in the absence of α‐synuclein pathology.^1^ Consistent with previous observations that hyposmia is a strong predictor of an underlying α‐synuclein pathology,^30^ both PD patients with hyposmia below the 10th percentile for whom CSF was available showed positive SAA results, whereas SAA of the prodromal individual without hyposmia was negative. Some genetic PD forms do not consistently show Lewy body pathology in the brain and positive SAA in the CSF, which is mainly observed for patients with pathogenic variants in the autosomal recessive genes *PRKN* and *PINK1*, but also in PARK‐*LRRK2* to varying degrees.^30^, ^31^ The proportion of positive SAAs in CSF was reported to be 67.5% in PARK‐*LRRK2* ^30^ and was especially present in patients or prodromal individuals with hyposmia, in keeping with the findings in our PARK‐*RAB32* patients. In our sample, 10 of 11 individuals exhibited hyposmia, which may suggest that PARK‐*RAB32* is associated with a more prominent α‐synuclein pathology compared to PARK‐*LRRK2*, where hyposmia—and the associated α‐synuclein pathology—is observed less frequently. Our findings also reveal possible pathophysiological implications, as RAB proteins are involved in the transport of α‐synuclein, among other functions, whereby a defect can lead to maldistribution and ultimately to the promotion of aggregation.^32^ It is important to note that the lack of α‐synuclein identification might also be related to its method's constraints, as recent investigations have revealed that additional forms of α‐synuclein pathology, such as oligomers, can be present in the brain tissue of Lewy‐body negative *LRRK2* patients.^33^, ^34^

**FIG. 3:**
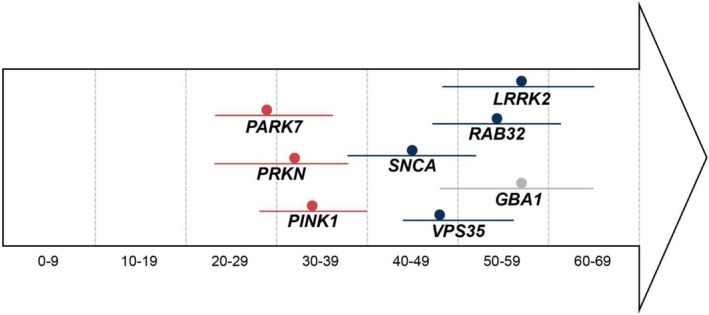
Comparison of the median age at onset (and interquartile ranges) for patients with different genetic forms of Parkinson's disease (PD), adapted from Vollstedt *et al*.^35^ (*Mov Disord*). red, autosomal recessive inherited genes (*PRKN, PINK1, PARK7*); blue, autosomal dominantly inherited genes (*LRRK2, SNCA, VPS35, RAB32*); gray, *GBA1*. [Color figure can be viewed at wileyonlinelibrary.com]

The median AAO of PARK‐*RAB32* fell between that of PARK‐LRRK2 and autosomal recessive PD forms (Fig. 3). However, we found a wide range of AAO from 31 to 82 years in the literature, calling for further investigations of the variable expressivity of the disease and modifying genetic and environmental factors. This observation should also be validated in larger cohorts and longitudinal studies of unaffected individuals with the pathogenic *RAB32* variant. Given the autosomal dominant inheritance pattern, the sex distribution in PARK‐*RAB32* is more balanced compared to idiopathic PD, where a male predominance is typically observed.

Remarkably, even in this newly discovered monogenic form of PD, the systematic literature search revealed a high proportion of unreported phenotypic features, especially non‐motor symptoms. In our deeply phenotyped cohort, we identified a remarkably high prevalence of symptoms such as hyposmia, depression, anxiety, or autonomic dysfunction through targeted testing, highlighting the importance of detailed phenotyping and systematic reporting. The most frequently reported non‐motor symptom was cognitive impairment, present in 36.6% of the individuals, which seems to be comparable to other autosomal dominantly inherited forms of PD (Table 2). In addition, no motor scales were reported (except for one person), which makes it difficult to draw conclusions about the severity and the individual course of the disease. A high proportion of missing data has also been found in previous MDSGene reviews for other monogenic forms of PD^10^, ^12^, ^36^, ^37^ and calls for systematic reporting of phenotype–genotype relationships. Furthermore, longitudinal data and further analysis of prodromal carriers are currently lacking to better understand disease progression. However, it should be noted that the *RAB32* cohort described in this study contains only a limited number of individuals.

Family history was negative in 26%, and pedigree analysis shows that some generations in larger families contain unaffected elderly individuals, indicating reduced disease penetrance. This is also known for individuals with PARK‐*LRRK2*. Polygenic modulation has already been shown to be a mechanism influencing penetrance among individuals carrying the *LRRK2* p.G2019S variant and may also play a role in PARK‐*RAB32*.^38^

In our cohort, the common haplotype was confirmed as previously described. The p.Ser71Arg variant predominantly occurs in patients from Southern Europe and North Africa (Fig. S1), suggesting a population‐specific origin and distribution of the variant. It has not been detected in large Asian PD cohorts,^39^, ^40^ nor in smaller cohorts from Africa (n = 353), Ashkenazi Jewish (n = 1105), or Latino and Indigenous people of America (n = 87)^41^; from Spain (n = 1,209 individuals)^40^ or Germany (n = 774 patients with PD).^42^ Further investigations, also of potential additional *RAB32* variants in diverse populations, are needed to better understand the distribution of PARK‐*RAB32* and its distinct pathophysiology, particularly in the context of emerging targeted therapeutic strategies.

In conclusion, we (1) identify the presence of α‐synuclein seeding and apply the recent SynNeurGe criteria to PARK‐*RAB32*; (2) demonstrate non‐motor signs to be an important part of PD linked to *RAB32*; and (3) provide the first MDSGene systematic literature review of PARK‐*RAB32*.

## Author Roles

(1) Research project: A. Conception, B. Organization, C. Execution; (2) Statistical Analysis: A. Design, B. Execution, C. Review and Critique; (3) Manuscript: A. Writing of the First Draft, B. Review and Critique.

T.K.: 1B, 1C, 2A, 2B, 2C, 3A

F.C.: 1B, 1C, 2B, 2C, 3B

M.B.: 1B, 1C, 2B, 2C, 3B

G.T.: 1B, 1C, 2B, 2C, 3B

F.V.: 1B, 1C, 2C, 3B

V.F.: 1B, 1C, 2C, 3B

E.M.V.: 1B, 1C, 2C, 3B

P.M.: 1B, 1C, 2C, 3B

M.A.: 1B, 1C, 2C, 3B

S.Z.: 1B, 1C, 2C, 3B

R.B.: 1B, 1C, 2C, 3B

M.M.: 1B, 1C, 2C, 3B

A.D.F.: 1B, 1C, 2C, 3B

E.M.: 1B, 1C, 2C, 3B

L.S.: 1B, 1C, 2B, 2C, 3B

N.G.: 1B, 1C, 2B, 2C, 3B

C.S.: 1B, 1C, 2C, 3B

M.B.: 1B, 1C, 2B, 2C, 3B

C.G.: 1B, 1C, 2C, 3B

C.Bl.: 1B, 1C, 2C, 3B

J.T.: 1B, 1C, 2C, 3B

M.R.: 1B, 1C, 2B, 2C, 3B

K.L.: 1B, 1C, 2C, 3B

C.Be.: 1B, 1C, 2B, 2C, 3B

P.B.: 1B, 1C, 2C, 3B

N.B.: 1B, 1C, 2B, 2C, 3B

C.K.: 1A, 1B, 2C, 3B

## Conflicts of Interest

The authors report no conflicts of interest regarding this manuscript. Financial disclosures for all authors are provided in Appendix A.

## Ethics Statement

The Ethics Committee of the University of Lübeck (Ratzeburger Allee 160, Building 70, 23,562 Lübeck) approved the study (2023‐105), and we obtained written consent from all examined participants. The study was implemented in accordance with the ethical standards set out in the 1964 Declaration of Helsinki and its subsequent amendments.

## Consent to Publication

All patients signed informed consent to publication of the data collected.

## Supporting information


**Supplementary Video S1.** L‐26617: The patient with a disease course of twelve years shows bradykinesia and a decrement in the finger tapping test, predominantly on the right side. The gait reveals moderate deceleration and hyperkinesia of both arms (especially the right arm).

**Supplementary Video S2.** L‐26618: The younger brother of L‐26617 has a disease course of seven years. The toe tapping shows interruption, lower frequency, and amplitude on the left side.

**Supplementary Video S3.** L‐26631: The patient, who has been affected for twenty years, has significant postural disturbances and is wheelchair dependent. Finger tapping and toe tapping show moderate bradykinesia.

**Supplementary Video S4.** L‐26913: The patient developed parkinsonism at the age of 46 years. Now, seven years later, she experiences frequent motor fluctuations. The finger tapping shows bradykinesia and a decrement, predominantly of the left side.

**Supplementary Video S5.** L‐26638: The individual (daughter of L‐26631) with subtle motor signs has hypomimia, bradykinesia in the finger tapping test predominantly on the left side, reduced arm swing primarily on the right side, and a delayed shoulder shrug on the right side.

**Supplementary Video S6.** L‐27022: The individual (son of L‐27021) is tested negative for the RAB32 p.Ser71Arg variant. He has an interruption, bradykinesia, and a decrement in the toe tapping test on the left side, as well as reduced arm swing, asymmetry, and delayed shoulder shrug on the right side.

**Data S1.** Supporting Information.
**Supplementary Table S1.** Search term for the literature search for *RAB32* on PubMed.
**Supplementary Table S2.** Comparison of two offspring of patients with PARK‐*RAB32*.
**Supplementary Table S3.** Genetic ancestry of patients with PARK‐*RAB32* and WGS data available (n = 5).
**Supplementary Table S4. Proportion of cognitive decline** in different monogenic PD forms (according to MDSGene; www.mds.gene.org).
**Supplementary Table S5. Brief reports of structural MR imaging** performed on individuals with PARK‐*RAB32*, if applicable.
**Supplementary Figure S1.** Country of origin of patients (or their parents) with PARK‐*RAB32* (self‐reported in 70.5% of all described individuals).
**Supplementary Figure S2 (A)‐(G).** Pedigrees of all investigated patients with a positive family history.
**Supplementary Figure S3.** Representative DaTSCAN Images of patients with PARK‐*RAB32*.

## Data Availability

The data generated here is available via the GP2 (https://gp2.org). Specifically, these are Tier 1/Tier 2 data from GP2 release R10. Tier 1 data can be accessed by completing a form on the Accelerating Medicines Partnership in Parkinson's Disease (AMP‐PD) website (https://amp-pd.org/register-for-amp-pd). Tier 2 data access requires approval and a Data Use Agreement signed by your institution. All other data used can be found on https://www.mdsgene.org/.

## Appendix A

**Teresa Kleinz**

**Table:**

Stock Ownership in medically‐related fields	None
Consultancies	None
Advisory Boards	None
Partnerships	None
Honoraria	None
Grants	Global Parkinson's Genetics Program (GP2)
Intellectual Property Rights	None
Expert Testimony	None
Employment	Institute of Neurogenetics, University of Lübeck, Lübeck, Germany and Section for Movement Disorders, Department of Neurology, University Hospital of Schleswig‐Holstein, Lübeck, Germany
Contracts	None
Royalties	None
Other	None

**Francesco Cavallieri**

**Table:**

Stock Ownership in medically‐related fields	None
Consultancies	None
Advisory Boards	None
Partnerships	None
Honoraria	Bial
Grants	None
Intellectual Property Rights	None
Expert Testimony	None
Employment	Neuromotor and Rehabilitation Department, Neurology Unit, Azienda USL‐IRCCS Di Reggio Emilia, Reggio Emilia, Italy.
Contracts	None
Royalties	None
Other	None

**Max Borsche**

**Table:**

Stock Ownership in medically‐related fields	None
Consultancies	None
Advisory Boards	None
Partnerships	None
Honoraria	Ipsen Pharma, Bial Germany
Grants	German Research Foundation (DFG)‐funded Clinician Scientist School, University of Lübeck; Else Kröner‐Fresenius‐Stiftung
Intellectual Property Rights	None
Expert Testimony	None
Employment	Institute of Neurogenetics, University of Lübeck, Lübeck, Germany and Section for Movement Disorders, Department of Neurology, University Hospital of Schleswig‐Holstein, Lübeck, Germany
Contracts	None
Royalties	None
Other	None

**Giulia Toschi**

**Table:**

Stock Ownership in medically‐related fields	None
Consultancies	None
Advisory Boards	None
Partnerships	None
Honoraria	None
Grants	None
Intellectual Property Rights	None
Expert Testimony	None
Employment	Neuromotor and Rehabilitation Department, Neurology Unit, Azienda USL‐IRCCS Di Reggio Emilia, Reggio Emilia, Italy.
Contracts	None
Royalties	None
Other	None

**Franco Valzania**

**Table:**

Stock Ownership in medically‐related fields	None
Consultancies	None
Advisory Boards	None
Partnerships	none
Honoraria	Bial
Grants	None
Intellectual Property Rights	None
Expert Testimony	None
Employment	Neuromotor and Rehabilitation Department, Neurology Unit, Azienda USL‐IRCCS Di Reggio Emilia, Reggio Emilia, Italy.
Contracts	None
Royalties	None
Other	None

**Valentina Fioravanti**

**Table:**

Stock Ownership in medically‐related fields	None
Consultancies	None
Advisory Boards	None
Partnerships	None
Honoraria	None
Grants	None
Intellectual Property Rights	None
Expert Testimony	None
Employment	Neuromotor and Rehabilitation Department, Neurology Unit, Azienda USL‐IRCCS Di Reggio Emilia, Reggio Emilia, Italy.
Contracts	None
Royalties	None
Other	None

**Enza Maria Valente**

**Table:**

Stock Ownership in medically‐related fields	None
Consultancies	None
Advisory Boards	None
Partnerships	None
Honoraria	None
Grants	Global Parkinson's Genetics Program (GP2), Telethon Italy, Cariplo Foundation, Italian Ministry of Health
Intellectual Property Rights	None
Expert Testimony	None
Employment	IRCCS C. Mondino Foundation, Pavia, Italy and Department of Molecular Medicine, University of Pavia, Pavia, Italy
Contracts	None
Royalties	None
Other	None

**Pierfrancesco Mitrotti**

**Table:**

Stock Ownership in medically‐related fields	None
Consultancies	None
Advisory Boards	None
Partnerships	None
Honoraria	None
Grants	None
Intellectual Property Rights	None
Expert Testimony	None
Employment	IRCCS C. Mondino Foundation, Pavia, Italy and Department of Brain and Behavioral Sciences, University of Pavia, Pavia, Italy
Contracts	None
Royalties	None
Other	None

**Micol Avenali**

**Table:**

Stock Ownership in medically‐related fields	None
Consultancies	None
Advisory Boards	None
Partnerships	None
Honoraria	None
Grants	Italian Ministry of Health
Intellectual Property Rights	None
Expert Testimony	None
Employment	IRCCS C. Mondino Foundation, Pavia, Italy and Department of Brain and Behavioral Sciences, University of Pavia, Pavia, Italy
Contracts	None
Royalties	None
Other	None

**Simone Zittel**

**Table:**

Stock Ownership in medically‐related fields	None
Consultancies	None
Advisory Boards	Biogen
Partnerships	None
Honoraria	Merz Pharmaceuticals
Grants	None
Intellectual Property Rights	None
Expert Testimony	None
Employment	Department of Neurology, University Medical Center Hamburg‐Eppendorf, Hamburg, Germany.
Contracts	None
Royalties	None
Other	None

**Rommi Born**

**Table:**

Stock Ownership in medically‐related fields	None
Consultancies	None
Advisory Boards	None
Partnerships	None
Honoraria	None
Grants	None
Intellectual Property Rights	None
Expert Testimony	None
Employment	Klinikum Hanau GmbH, Department of Neurology, Hanau, Germany
Contracts	None
Royalties	None
Other	None

**Michele Matarazzo**

**Table:**

Stock Ownership in medically‐related fields	Biogen, United Health
Consultancies	None
Advisory Boards	None
Partnerships	None
Honoraria	Zambon, Bial, Insightec
Grants	Michael J Fox Foundation
Intellectual Property Rights	None
Expert Testimony	None
Employment	Centro Integral de Neurociencias Abarca Campal, Hospital Universitario HM Puerta Del Sur, HM Hospitales, Madrid, Spain.
Contracts	None
Royalties	None
Other	None

**Alessio Di Fonzo**

**Table:**

Stock Ownership in medically‐related fields	None
Consultancies	None
Advisory Boards	Sanofi
Partnerships	None
Honoraria	Sanofi, Zambon
Grants	None
Intellectual Property Rights	None
Expert Testimony	None
Employment	IRCCS Foundation Ca′ Granda Ospedale Maggiore Policlinico, Dino Ferrari Center, Neuroscience Section, Department of Pathophysiology and Transplantation, University of Milan, Milan, Italy.
Contracts	None
Royalties	None
Other	None

**Edoardo Monfrini**

**Table:**

Stock Ownership in medically‐related fields	None
Consultancies	None
Advisory Boards	None
Partnerships	None
Honoraria	None
Grants	None
Intellectual Property Rights	None
Expert Testimony	None
Employment	IRCCS Foundation Ca′ Granda Ospedale Maggiore Policlinico, Dino Ferrari Center, Neuroscience Section, Department of Pathophysiology and Transplantation, University of Milan, Milan, Italy.
Contracts	None
Royalties	None
Other	None

**Mandy Radefeldt**

**Table:**

Stock Ownership in medically‐related fields	None
Consultancies	None
Advisory Boards	None
Partnerships	None
Honoraria	None
Grants	None
Intellectual Property Rights	None
Expert Testimony	None
Employment	Centogene GmbH, Rostock, Germany
Contracts	None
Royalties	None
Other	None

**Letizia Santinelli**

**Table:**

Stock Ownership in medically‐related fields	None
Consultancies	None
Advisory Boards	None
Partnerships	None
Honoraria	None
Grants	None
Intellectual Property Rights	None
Expert Testimony	None
Employment	Institute of Neurogenetics, University of Lübeck, Lübeck, Germany
Contracts	None
Royalties	None
Other	None

**Norman Griebner**

**Table:**

Stock Ownership in medically‐related fields	None
Consultancies	None
Advisory Boards	None
Partnerships	None
Honoraria	None
Grants	None
Intellectual Property Rights	None
Expert Testimony	None
Employment	Section for Movement Disorders, Department of Neurology, University Hospital of Schleswig‐Holstein, Lübeck, Germany.
Contracts	None
Royalties	None
Other	None

**Cholpon Shambetova**

**Table:**

Stock Ownership in medically‐related fields	None
Consultancies	None
Advisory Boards	None
Partnerships	None
Honoraria	None
Grants	Global Parkinson's Genetics Program (GP2), Aligning Science Across Parkinson's (ASAP), Michael J. Fox Foundation for Parkinson's Research (MJFF)
Intellectual Property Rights	None
Expert Testimony	None
Employment	Kyrgyz State Medical Academy, Kyrgyzstan and Institute of Neurogenetics, University of Lübeck, Lübeck, Germany
Contracts	None
Royalties	None
Other	None

**Max Brand**

**Table:**

Stock Ownership in medically‐related fields	None
Consultancies	None
Advisory Boards	None
Partnerships	None
Honoraria	None
Grants	None
Intellectual Property Rights	None
Expert Testimony	None
Employment	Institute of Neurogenetics, University of Lübeck, Lübeck, Germany
Contracts	None
Royalties	None
Other	None

**Carolin Gabbert**

**Table:**

Stock Ownership in medically‐related fields	None
Consultancies	None
Advisory Boards	None
Partnerships	None
Honoraria	None
Grants	Global Parkinson's Genetics Program (GP2)
Intellectual Property Rights	None
Expert Testimony	None
Employment	Institute of Neurogenetics, University of Lübeck, Lübeck, Germany
Contracts	None
Royalties	None
Other	None

**Cornelis Blauwendraat**

**Table:**

Stock Ownership in medically‐related fields	None
Consultancies	None
Advisory Boards	None
Partnerships	None
Honoraria	None
Grants	Michael J. Fox Foundation for Parkinson's Research, Aligning Science Across Parkinson's Initiative
Intellectual Property Rights	None
Expert Testimony	None
Employment	Center for Alzheimer's and Related Dementias (CARD), National Institute on Aging and National Institute of Neurological Disorders and Stroke, National Institutes of Health, Bethesda, MD, USA and Laboratory of Neurogenetics, National Institute on Aging, National Institutes of Health, Bethesda, MD, USA
Contracts	None
Royalties	None
Other	Intramural Research Program of the National Institute on Aging

**Joanne Trinh**

**Table:**

Stock Ownership in medically‐related fields	None
Consultancies	Acurex
Advisory Boards	None
Partnerships	None
Honoraria	None
Grants	DFG (FOR2488), Heisenberg Grant
Intellectual Property Rights	None
Expert Testimony	None
Employment	Institute of Neurogenetics, University of Lübeck, Lübeck, Germany
Contracts	None
Royalties	Elsevier publisher
Other	None

**Katja Lohmann**

**Table:**

Stock Ownership in medically‐related fields	None
Consultancies	None
Advisory Boards	None
Partnerships	None
Honoraria	None
Grants	German Research Foundation, MJFF (GP2) & Dystonia Medical Research Foundation
Intellectual Property Rights	None
Expert Testimony	None
Employment	Institute of Neurogenetics, University of Lübeck, Lübeck, Germany
Contracts	None
Royalties	Elsevier publisher
Other	None

**Christian Beetz**

**Table:**

Stock Ownership in medically‐related fields	None
Consultancies	None
Advisory Boards	None
Partnerships	None
Honoraria	None
Grants	The Michael J. Fox Foundation
Intellectual Property Rights	None
Expert Testimony	None
Employment	Centogene GmbH, Rostock, Germany.
Contracts	None
Royalties	None
Other	None

**Peter Bauer**

**Table:**

Stock Ownership in medically‐related fields	None
Consultancies	None
Advisory Boards	None
Partnerships	None
Honoraria	None
Grants	None
Intellectual Property Rights	None
Expert Testimony	None
Employment	Centogene GmbH, Rostock, Germany.
Contracts	none
Royalties	none
Other	none

**Norbert Brüggemann**

**Table:**

Stock Ownership in medically‐related fields	None
Consultancies	None
Advisory Boards	Zambon
Partnerships	None
Honoraria	Abbvie, Esteve, Ipsen, Merz, Takeda, Teva, Zambon
Grants	DFG (BR4328.2–1, GRK1957), Michael J Fox Foundation, Collaborative Center for X‐linked Dystonia‐Parkinsonism EU Joint Programme – Neurodegenerative Disease Research (JPND)
Intellectual Property Rights	None
Expert Testimony	None
Employment	University of Lübeck and Medical University Center Schleswig‐Holstein
Contracts	None
Royalties	None
Other	None

**Christine Klein**

**Table:**

Stock Ownership in medically‐related fields	None
Consultancies	Centogene, Takeda
Advisory Boards	Retromer Therapeutics
Partnerships	None
Honoraria	Desitin and Bial
Grants	DFG, MJFF
Intellectual Property Rights	None
Expert Testimony	None
Employment	University of Lübeck, Lübeck, Germany and Section for Movement Disorders, Department of Neurology, University Hospital of Schleswig‐Holstein, Lübeck, Germany
Contracts	None
Royalties	Oxford University Press
Other	None
