# Supplementary material for: RAB32 ‐Linked Parkinson's Disease: Deep Phenotyping, MDSGene Literature Review, and Application of SynNeurGe Criteria
Source: Mov Disord. 2025 Oct 17;40(12):2746–69. doi: 10.1002/mds.70037 (PMC12710152; doi:10.1002/mds.70037)
Supplement: Supplementary file 7 — Data S1. Supporting Information. Supplementary Table S1. Search term for the literature search for RAB32 on PubMed. Supplementary Table S2. Comparison of two offspring of patients with PARK‐RAB32. Supplementary Table S3. Genetic ancestry of patients with PARK‐RAB32 and WGS data available (n = 5). Supplementary Table S4. Proportion of cognitive decline in different monogenic PD forms (according to MDSGene; www.mds.gene.org). Supplementary Table S5. Brief reports of structural MR imaging performed on individuals with PARK‐RAB32, if applicable. Supplementary Figure S1. Country of origin of patients (or their parents) with PARK‐RAB32 (self‐reported in 70.5% of all described individuals). Supplementary Figure S2 (A)‐(G). Pedigrees of all investigated patients with a positive family history. Supplementary Figure S3. Representative DaTSCAN Images of patients with PARK‐RAB32. [file MDS-40-2746-s007.docx]

**Supplementary Material**

**Supplementary Table 1.** Search term for the literature search for *RAB32* on PubMed

| *RAB32* | (ataxia OR ataxic OR cerebellar OR channelopathy OR dystonia OR dystonic OR parkinson* OR paroxysmal movement OR tremor OR myoclon* OR chorea OR choreo* OR choreatic OR spastic paraplegia OR spastic paraparesis OR HSP OR Strümpell* OR hyperkinetic OR “movement disorder” OR dyskinesia OR dyskinetic) AND (*RAB32*) AND ("english"[Language]) |
| --- | --- |

**Supplementary Table 2.** Comparison of two offspring of patients with PARK-*RAB32*

| Participants (local ID, sex) | L-26638, female | L-27022, male |
| --- | --- | --- |
| Index patient of the family | L-26631 | L-26921 |
| Genetic status | *RAB32* (Ser71Arg) positive (het) | *RAB32* (Ser71Arg) negative |
| Age at examination | 58 years | 40 years |
| UPSIT result | normal | normal |
| RBDSQ score | 5 points | 5 points |
| Motor examination findings | Mild hypomimia and lateralized bradykinesia, including reduced arm swing and delayed shoulder shrug on the right side* | Reduced arm swing and delayed shoulder shrug on the left side, bradykinesia, and interruptions in the foot-tapping test* |

*Supplementary Videos

UPSIT, University of Pennsylvania Smell Identification Test; RBDSQ, REM sleep behavior disorder screening questionnaire

| **Local ID** | **ctry** | **Geo-region** | **AHG** | **Afri-can** | **Cauca-sian** | **Dravi-dian** | **East-**  **afri-can** | **Near-**  **East** | **North-**  **euro-pean** | **Sahul** | **Sino-**  **tibetan** | **South-**  **euro-pean** | ***** |
| --- | --- | --- | --- | --- | --- | --- | --- | --- | --- | --- | --- | --- | --- |
| L-26617 | ITA | Europe | 0 | 0 | 0,1997 | 0,1201 | 0,0557 | 0,1806 | 0,1126 | 0,0566 | 0,0611 | 0,2137 | 0 |
| L-26618 | ITA | Europe | 0 | 0,0694 | 0,2385 | 0,1011 | 0 | 0,1919 | 0,1159 | 0,0507 | 0,0608 | 0,1718 | 0 |
| L-26631 | GER | Europe | 0,0402 | 0 | 0,148 | 0,112 | 0 | 0,0825 | 0,2734 | 0,0714 | 0,0509 | 0,2216 | 0 |
| L-26753 | ITA | Europe | 0 | 0 | 0,1845 | 0,1241 | 0,0531 | 0,1713 | 0,1596 | 0,0473 | 0,0516 | 0,2085 | 0 |
| L-26915 | ITA | Europe | 0 | 0 | 0,1974 | 0,119 | 0,0598 | 0,1453 | 0,2035 | 0,0661 | 0 | 0,209 | 0 |

**Supplementary Table 3**. Genetic ancestry of patients with PARK-*RAB32* and WGS data available (n=5)

***Amerindian, Arctic, Austronesian, EA, Northindian, Paleosiberian, SEA, Saami, Samaritan, Samoedic, Siberian, Uralic**

ctry, country; ITA, Italy; Ger, Germany

**Supplementary Table 4. Proportion of cognitive decline** in different monogenic PD forms (according to MDSGene; www.mds.gene.org)

| **Gene** | **Proportion of cognitive decline [%]** | **Total number of patients with reported cognitive decline** |
| --- | --- | --- |
| LRRK2 | 26.7% | n=470 |
| VPS35 | 44.4% | n=9 |
| SNCA | 70.2% | n=94 |
| RAB32 | 36.6%* (35.3% only literature) | n=45* (n=34 only literature) |
| GBA1 | 62% | n=276 |
| PRKN | 19.5% | n=133 |
| PINK1 | 30% | n=110 |
| PARK7 | 35.7% | n=14 |

*including the investigated *RAB32* cohort

**Supplementary Table 5. Brief reports of structural MR imaging** performed on individuals with PARK-*RAB32*, if applicable

| **Patient ID** | **Written report of the structural MRI of the brain** |
| --- | --- |
| L-26617 | Regular presentation of the brain tissue and cerebrospinal fluid spaces. |
| L-26618 | Minimal microangiopathic alterations. Otherwise, normal presentation of the brain tissue and cerebrospinal fluid spaces |
| L-26753 | Regular presentation of the brain tissue and cerebrospinal fluid spaces. |
| L-26809 | Single medullary gliosis, partially post-inflammatory in appearance. Partially calcified falx myoma. |
| L-26913 | Age-appropriate presentation of the brain tissue and cerebrospinal fluid spaces. |
| L-27021 | Suspected lacunar infarct residuum in the right caudate nucleus, DD locally dilated Virchow-Robin perivascular space. Medullary defects on both sides, supratentorially and in the pons, with primarily expression of a moderate cerebral microangiopathy. Otherwise, normal visualization of the brain tissue and cerebrospinal fluid spaces. |
| L-26638 | Minor cerebral microangiopathy, also questionable with pontine manifestation, DD artificial. Otherwise, normal visualization of the brain tissue and cerebrospinal fluid spaces. |

DD, Differential diagnosis


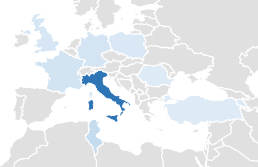

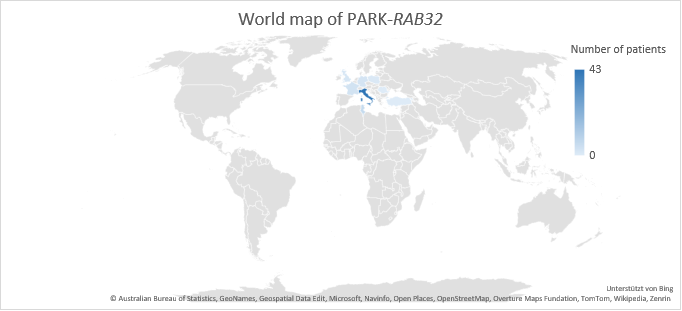


**Supplementary Figure 1.** Country of origin of patients (or their parents) with PARK-*RAB32* (self-reported in 70.5% of all described individuals)


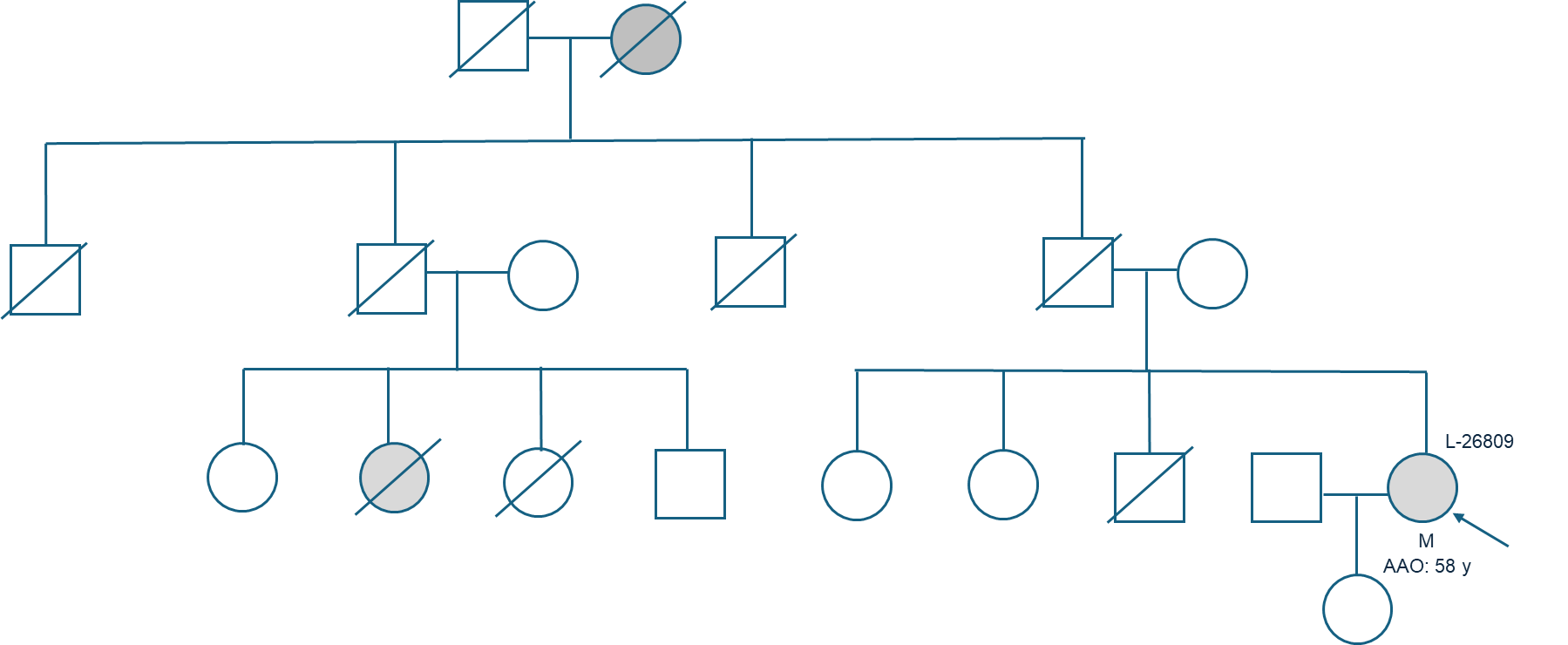


1. **ARM I**, L-26809 (Index), was investigated.

The patient’s paternal grandmother was affected by PD with an AAO of 42 years and died at the age of 66 years. A paternal cousin had an estimated AAO of 55 years and died at 75. The patient’s father died at the age of 79 years without Parkinsonian signs.


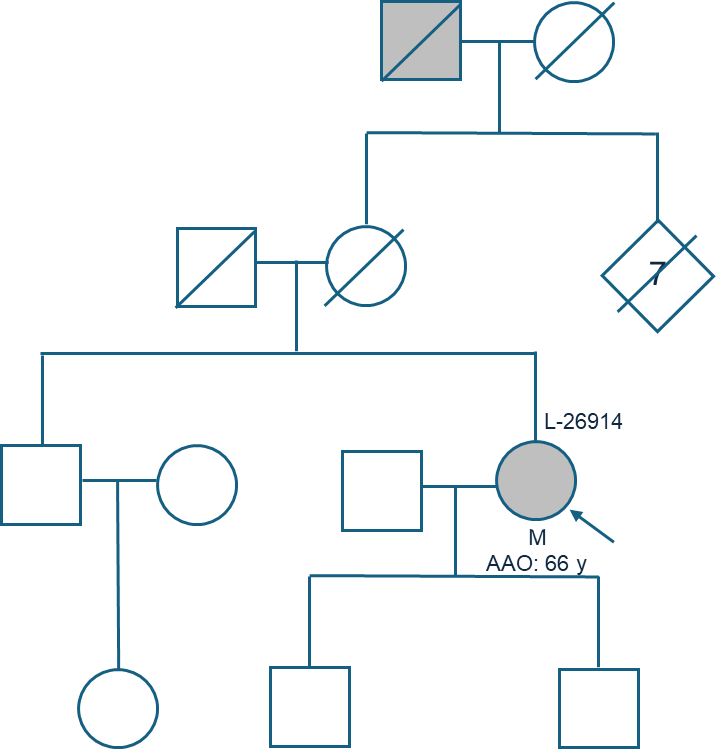


1. **ITA IV**, L-26914 (Index) was investigated.

The patient's mother died at the age of 48, and they had seven siblings; the patient did not recall any diagnosis of Parkinson's in this generation. The patient's grandfather had tremor and bradykinesia. Neither of the patients' sons has been tested.


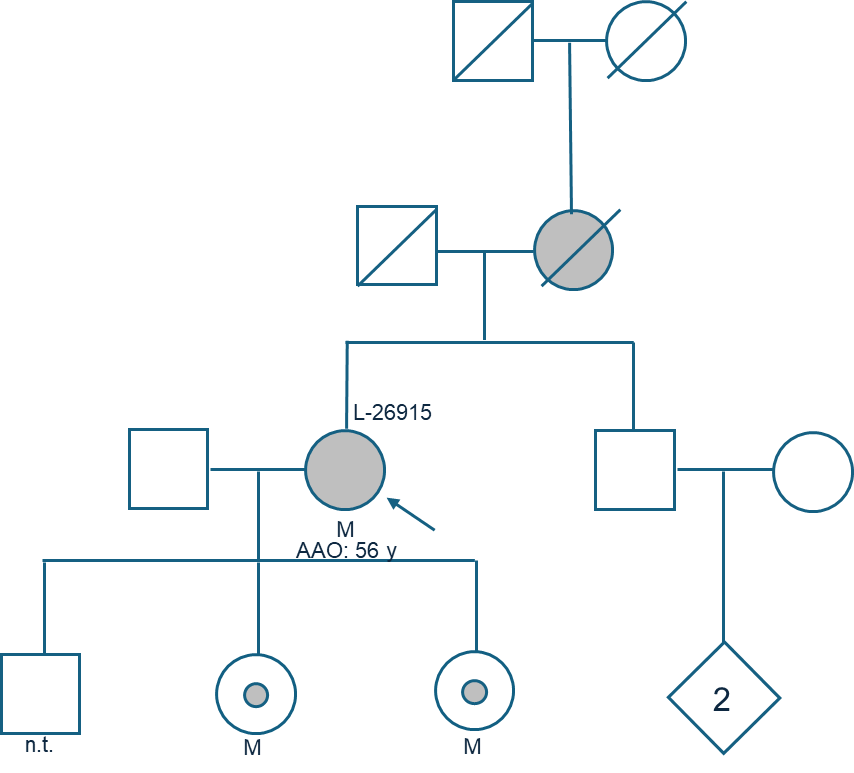


1. **ITA V**, L-26915 is the Index patient.

She recalled a PD diagnosis of her mother at a very late age. Both daughters of the patients are heterozygous carriers of *RAB32* Ser71Arg but have not yet been examined. The patient’s son was not tested.


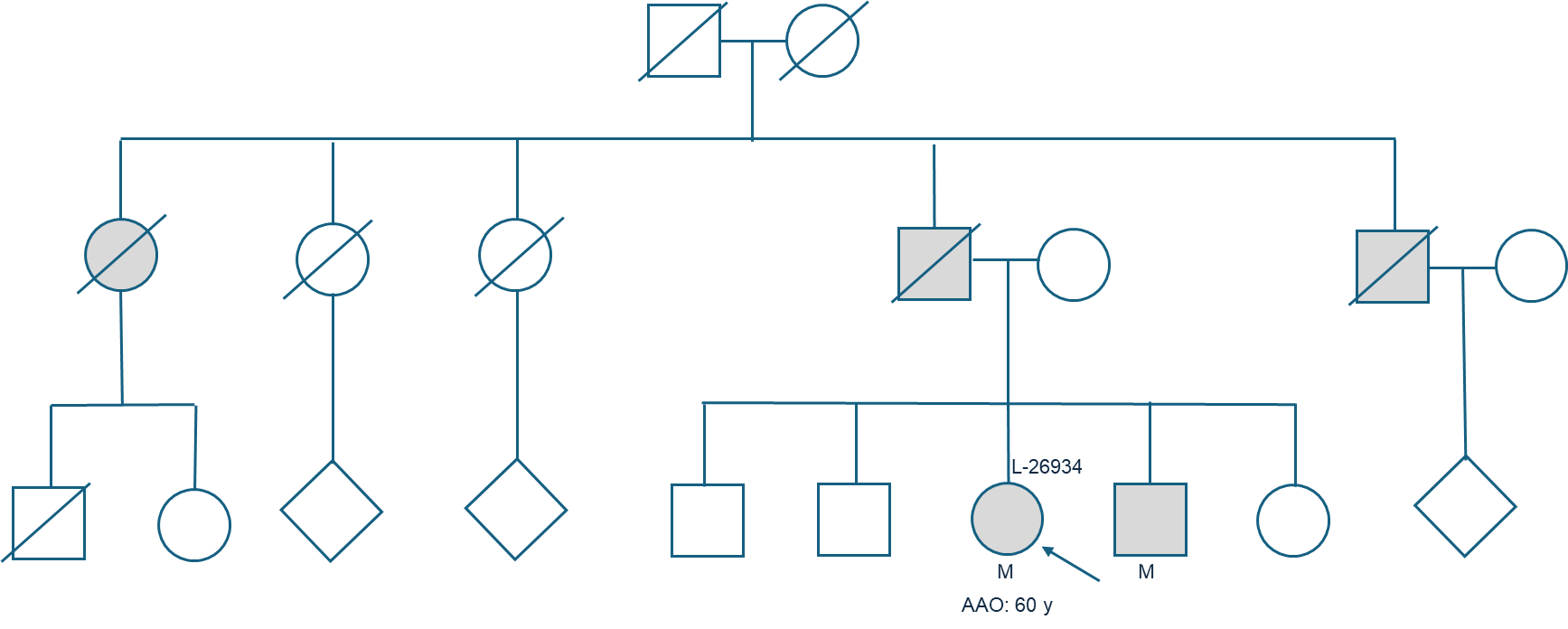


1. **ITA VI**, L-26934 (Index), was investigated.

The patient’s brother was diagnosed with PD very recently (AAO: 60 years), and the *RAB32* p.Ser71Arg variant was confirmed by genetic testing. The patient’s father (AAO ~60 years), one uncle (AAO: 40 years), and one aunt (AAO unknown) from the paternal side were also affected by PD. The paternal grandfather died at the age of 40 years, and the grandmother at the age of 50 years without Parkinsonian signs.


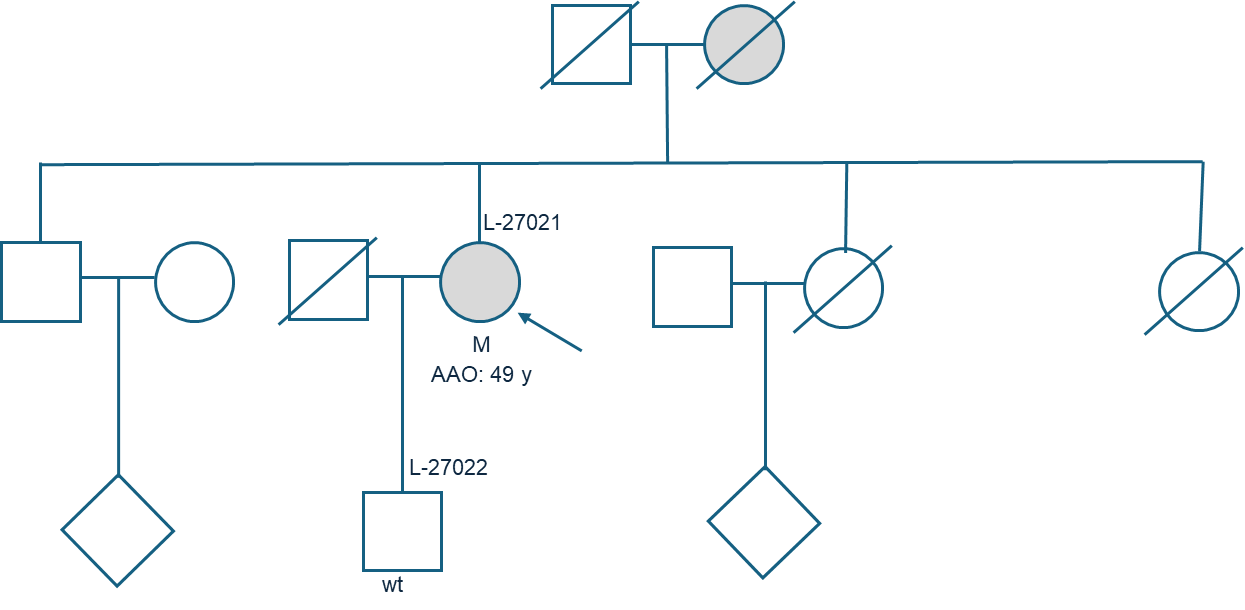


1. **ESP-I**, L-27021 (Index), and her son (wt for RAB32 p.Ser71Arg) were investigated.

The patient recalled a diagnosis of tremor and slow movements in her mother. The patient’s younger sisters died at the ages of about 40 and 30 years.

**Supplementary Figure 2 (A)-(G).** Pedigrees of all investigated patients with a positive family history.

PD, Parkinson’s Disease; AAO, age at onset; M, confirmed mutant; wt, wildtype; n.t., not tested


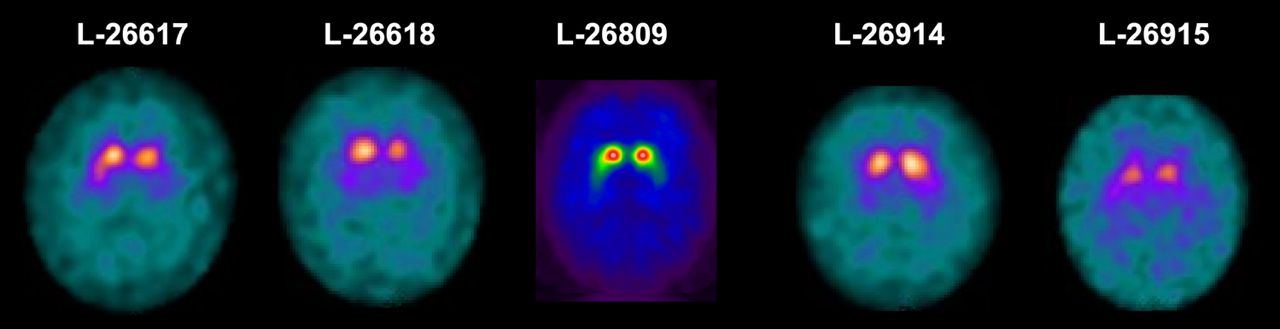


**Supplementary Figure 3.** Representative DaTSCAN Images of patients with PARK-*RAB32*

**Supplementary videos legend**

1) L-26617: The patient with a disease course of twelve years shows bradykinesia and a decrement in the finger tapping test, predominantly on the right side. The gait reveals moderate deceleration and hyperkinesia of both arms (especially the right arm).

2) L-26618: The younger brother of L-26617 has a disease course of seven years. The toe tapping shows interruption, lower frequency, and amplitude on the left side.

3) L-26631: The patient, who has been affected for twenty years, has significant postural disturbances and is wheelchair dependent. Finger tapping and toe tapping show moderate bradykinesia.

4) L-26913: The patient developed parkinsonism at the age of 46 years. Now, seven years later, she experiences frequent motor fluctuations. The finger tapping shows bradykinesia and a decrement, predominantly of the left side.

5) L-26638: The individual (daughter of L-26631) with subtle motor signs has hypomimia, bradykinesia in the finger tapping test predominantly on the left side, reduced arm swing primarily on the right side, and a delayed shoulder shrug on the right side.

6) L-27022: The individual (son of L-27021) is tested negative for the RAB32 p.Ser71Arg variant. He has an interruption, bradykinesia, and a decrement in the toe tapping test on the left side, as well as reduced arm swing, asymmetry, and delayed shoulder shrug on the right side.
